# Tele-Neurorehabilitation During the COVID-19 Pandemic: Implications for Practice in Low- and Middle-Income Countries

**DOI:** 10.3389/fneur.2021.667925

**Published:** 2021-10-05

**Authors:** Abhishek Srivastava, Aishwarya Swaminathan, Manigandan Chockalingam, Murali K. Srinivasan, Nirmal Surya, Partha Ray, Prasanna S. Hegde, Preetie Shetty Akkunje, Sanjivani Kamble, Sonal Chitnis, Sureshkumar Kamalakannan, Suvarna Ganvir, Urvashi Shah, The Indian Federation of Neurorehabilitation (IFNR) Research Task Force, Abhishek Srivastava

**Affiliations:** ^1^Centre for Physical Medicine and Rehabilitation, Kokilaben Dhirubhai Ambani Hospital and Medical Research Institute, Mumbai, India; ^2^School of Occupational Therapy, D. Y. Patil University, Navi Mumbai, India; ^3^National University of Ireland Galway, Galway, Ireland; ^4^Faculty of Occupational Therapy, Meenakshi Academy of Higher Education and Research, Chennai, India; ^5^Chairman Surya Neuro Centre Mumbai, President Indian Federation of Neurorehabilitation (IFNR), Mumbai, India; ^6^National Health Services England, The Walton Centre Liverpool & National Professor of Neurology, Liverpool, United Kingdom; ^7^Deglutology and Speech-Language Pathology, HCG Hospital, Bangalore, India; ^8^Department of Speech Pathology and Audiology, National Institute of Mental Health and Neurosciences, Bangalore, India; ^9^Dr. D. Y. Patil College of Physiotherapy, Dr. D. Y. Patil Vidyapeeth, Pune, India; ^10^Bharati Vidyapeeth (Deemed to be) University, School of Audiology Speech Language Pathology, Pune, India; ^11^South Asia Centre for Disability Inclusive Development and Research (SACDIR), Public Health Foundation of India, The Indian Institute of Public Health Hyderabad, Hyderabad, India; ^12^Department of Neuro Physiotherapy, Dr. Vitalrao Vikhe Patil Foundation's College of Physiotherapy, Ahmednagar, India; ^13^Department of Neurology, King Edward Memorial and Global Hospitals Mumbai, Mumbai, India

**Keywords:** neurorehabilitation, tele-rehabilitation system, neurological disability, COVID-19, pandemic (COVID-19), low- and middle-income countries

## Abstract

The importance of neurorehabilitation services for people with disabilities is getting well-recognized in low- and middle-income countries (LMICs) recently. However, accessibility to the same has remained the most significant challenge, in these contexts. This is especially because of the non-availability of trained specialists and the availability of neurorehabilitation centers only in urban cities owned predominantly by private healthcare organizations. In the current COVID-19 pandemic, the members of the Task Force for research at the Indian Federation of Neurorehabilitation (IFNR) reviewed the context for tele-neurorehabilitation (TNR) and have provided the contemporary implications for practicing TNR during COVID-19 for people with neurological disabilities (PWNDs) in LMICs. Neurorehabilitation is a science that is driven by rigorous research-based evidence. The current pandemic implies the need for systematically developed TNR interventions that is evaluated for its feasibility and acceptability and that is informed by available evidence from LMICs. Given the lack of organized systems in place for the provision of neurorehabilitation services in general, there needs to be sufficient budgetary allocations and a sector-wide approach to developing policies and systems for the provision of TNR services for PWNDs. The pandemic situation provides an opportunity to optimize the technological innovations in health and scale up these innovations to meet the growing burden of neurological disability in LMICs. Thus, this immense opportunity must be tapped to build capacity for safe and effective TNR services provision for PWNDs in these settings.

## Introduction

Neurological disorders are the leading causes of disability globally (1). During the past three decades, there is an absolute increase in people with neurological disability (PWND) by ~77.3% (2). A substantial proportion of this neurological disability burden are borne by low- and middle-income countries (LMICs) (3). Although there are several advances in the prevention and management of neurological disorders globally, factors such as demographic (increasing aging population) and epidemiological transitions (increasing non-communicable diseases) are consistently adding up to this burden (4).

The recent COVID-19 pandemic has tremendously halted most of the efforts toward combating the growing burden of neurological disorders, especially in LMICs (5). People with neurological disability (PWND) are unable to access neurorehabilitation services (5). Although access to such services was not available even during the pre-pandemic times, it is even harder to access during the COVID-19 pandemic (6). The shortage of specialists involved in neurorehabilitation, such as physiatrists, neurologists, rehabilitation nurses, physiotherapists, neuropsychologists, occupational therapists (OTs), speech and language pathologists, prosthetists and orthotists, and nutritionists, in LMICs has become even more acute in these settings (7). The neurorehabilitation specialists also experience an ambiguous situation during the pandemic due to the lack of specific guidelines to provide neurorehabilitation services for PWND (8).

Telerehabilitation has been perceived as a key innovation and an effective strategy to combat the existing pandemic situation and reduce the global burden of disability (9). Telecommunication technology has been a powerful tool to enhance the provision of health, education, and development services during the COVID-19 pandemic worldwide (10). It is also envisaged to be a game-changer in addressing the global burden of disability (9, 10). Even during the pre-pandemic times, these telecommunication technologies were substantially optimized to provide uninterrupted neurological rehabilitation services and care for PWNDs. They are popularly known as tele-neurorehabilitation (TNR) services (11). TNR is gaining considerable momentum globally in recent times (12). Though TNR services are well-organized and guided by good quality evidence in high-income countries (HICs), these services are yet to be systematically developed and tested for feasibility, acceptability, and effectiveness in LMICs (11, 13). In the current COVID-19 pandemic, the members of the Task Force for research at the Indian Federation of Neurorehabilitation (IFNR) reviewed the context for TNR and have provided the contemporary implications for practicing TNR during COVID-19 for PWNDs in LMICs. This critical reflection from the task force would potentially help to arrive at a policy or consensus for the provision of TNR services for PWNDs in LMICs.

### The Practice of TNR in LMICs

With the largest number of internet users in Latin America, Brazil has introduced, accelerated emergency regulations for provision of tele-rehabilitation, and remotely delivered interventions to promote access to rehabilitation (14). A review of home-based tele-rehabilitation services in Southeast Asian countries found that the completion rates of interventions accessed by people with disabilities have been ~80% in China and South Korea (15). A recent survey from the sub-continent (India) had showed close to 80% of the rehabilitation facilities with basic tele-rehabilitation infrastructure (16). However, tele-health and rehabilitation services are not available and are poorly utilized by the government primary care systems in many countries in the region of Sub-Saharan Africa (17).

The practice of tele-health and rehabilitation had been an emerging science in improving access to healthcare and rehabilitation globally, even before the COVID-19 pandemic (18). The current pandemic has enabled its growth manifold by default worldwide. Tele-health and rehabilitation have become inevitable to meet the demands of those who need continued support globally (19). There have been a wide range of interventions and rationale for TNR. However, the experiences and evidence for organized provision of TNR services are very limited in LMICs.

Most types of TNR services in LMICs are used for two primary purposes: 1. clinical assessment and 2. therapeutic rehabilitation (20). The practice of neurorehabilitation has become more rewarding with the introduction/facilitation of TNR practice during COVID times. Guidelines for remote prescribing in several LMICs have also created access to medicines and strengthen primary care during the pandemic (21). A substantial amount of patient referrals are currently handled remotely through TNR (22). Tele-consultations are seen as equally effective and efficient as the face-to-face interaction with the added advantage of avoiding unnecessary exposure to infections (23). PWNDs are assessed and treated in their actual living environment, which is highly encouraging for the patients. It also reduces the service cost, and it cuts down the cost of traveling to access rehabilitation (23, 24). Therapeutic progress is currently being well-documented because of the auto-digitization features of telecommunication technology (23, 24). Overall, access to rehabilitation has improved with the introduction of TNR services. PWNDs who cannot travel due to their disability and the lockdown restrictions can still access rehabilitation and care without any access barriers and opportunity cost. Both providers of neurorehabilitation and the consumers are continuing to adapt to deliver and access services through TNR, respectively, in the current pandemic situation.

### Implications for Practice of TNR in LMICs During the COVID-19 Pandemic

#### Basal Implications

Overall, there are several implications to evaluate PWNDs and provide specialized, comprehensive multidisciplinary neurorehabilitation through TNR services in LMICs (8, 25). A key aspect to remember is that there must not be any compromise on the objectivity of the assessment or evaluation and therapeutic approaches for the management of the needs of PWNDs. Neurorehabilitation is a science that is driven by rigorous research-based evidence. Therefore, assessing and providing neurorehabilitation services, whether provided in-person or using telerehabilitation, must not have any compromise on its objectivity and evidence for evaluation and treatment. The current pandemic implies the need for systematically developed TNR interventions that is evaluated for its feasibility and acceptability and that is informed by available evidence from LMICs.

It is essential to understand the implications of TNR as this would enable identification of effective strategies for comprehensive assessment and treatment that can meet the needs of PWNDs. Though a potential opportunity, TNR cannot entirely replace the actual ways of delivering neurological rehabilitation (26). Neurologists can consult patients, provide treatment, and prescribe medications and referrals as appropriate. Optimizing TNR services for consultations had been proven feasible in HICs before and during the pandemic times (10, 18). However, available resources such as adequate internet bandwidth, devices with required configurations, and information management systems for implementing TNR services need to be in place. For instance, close to 1/3 of the Brazilian population lack access to internet (27). Although the providers have access to basic tele-rehabilitation infrastructure like in India, the consumers need to have internet access to avail such services (16).

#### Physiatrist Perspectives

From a physiatrist's perspective, continuity of treatment and care is something that TNR services could seamlessly support. PWNDs need continued care, and that can be enhanced through multidisciplinary neurorehabilitation team consultations led by the physiatrist virtually (28). Many South-Asian countries including India had come up with national guidelines on this (28, 29). Singapore's guidelines were considered comparatively comprehensive (29). Assessment of basic vital parameters, neurological status, pain, sleep, energy, spasticity, bladder and bowel, and mobility status; functionality of tracheostomy, feeding tube, and urinary catheters; patient and caregiver education; appropriate instructions for providing basic support; and interventions to prevent secondary complications are feasible with trained manpower keeping in mind the nature and course of the disease. However, to operationalize this, PWNDs and their caregivers must be thoroughly educated about these aspects of TNR. The literacy and understanding about tele-rehabilitation and use of tele-communication technologies for rehabilitation has been poor among those PWNDs even before the pandemic in many LMICs especially in Sub-Saharan Africa (29, 30). Considering these aspects will be crucial to enhance TNR services and enable service providers to achieve neurorehabilitation goals in a realistic way within their environment.

#### Neuropsychological Perspectives

In this pandemic context, the neuropsychologist is expected to conduct neuropsychological evaluations and provide psychotherapy and cognitive rehabilitation for PWNDs through TNR (31, 32). However, it urges the understanding of its key implications. For instance, in Israel, the neuropsychological assessment services were postponed due to the pandemic with many people requiring such services put on a long waiting list. To combat this, the Israel government had developed remote solutions to meet the increasing demand for such services (33). Though these guidelines keep getting developed in many LMICs, some caveats need to be considered especially when it comes to administering standardized neuropsychological tests. There are significant differences between conducting a standardized neuropsychological evaluation in an ideal environment as compared to a virtual environment. Interpretation of the evaluations might differ and may not be the same while this is carried out virtually (33). Also, the neurorehabilitation team may have to rely substantially on the caregivers and patients to engage in the sessions proactively.

#### Physiotherapy Perspectives

Given the high demand for physiotherapy services in LMICs, neuro-physiotherapists are accustomed to evaluate the range of motion, strength, muscle tone, and endurance of PWNDs through eye-balling sometimes. They are also competent to conduct a thorough neurological examination, including cranial nerve testing through performance-based assessments. Therefore, it is feasible to assess neurological disability remotely through TNR services (34, 35). However, the provision of actual therapy or intervention using specialized techniques must be carried out with utmost safety considerations. During the provision of TNR physiotherapy services, patients and caregivers may not comprehend the instructions as they do this in-person, leading to serious untoward incidences. Hence, many HICs like Australia and the UK recommend developing highly competent inter-professional rehabilitation services as well as a frameworks or guidelines to enhance the provision of physiotherapy services remotely using technology (36).

#### Speech–Language Pathology Perspectives

Speech–language pathologists (SLPs) are one of the key neurorehabilitation professionals who are not easily available and accessible in LMICs (37). It was estimated that there were only 2,500 SLPs in India, which is an acute shortage of such key neurorehabilitation professionals delivering care (38). However, it is commonly perceived that provision of assessment and rehabilitation for patients with neurological and neurosurgical disorders presenting with safe swallowing, speech, language, and cognitive-communication dysfunctions are some of the key aspects to include during tele-practice by SLPs across all age group in LMICs (39). Use of hybrid methods could also be a potential strategy in the management of neurodevelopmental and acquired communication disorders, dysarthria, oropharyngeal dysphagia, and cognitive-communication disorders experienced by patients reporting in outpatient as well as in-patient settings (40). There are examples from certain countries like the Indian Speech and Hearing Association that had published the tele-practice guidelines and specific resource material for speech–language pathology and audiology services in India (41). However, it is well-known that even during the pre-pandemic times, it was estimated that only 10%-12% of SLPs tend to use tele-practice as a strategy for providing SLP services in India (38). A similar survey recently in Croatia had also estimated that only 3% of those SLPs surveyed had completed a formal training related to tele-practice in SLP services. Several consumers in the survey expressed the lack of equipment and trust on the effectiveness of tele-practice as the reasons for non-utilization of such services (42). Hence, organizing TNR services provided for swallowing and cognitive communication requires careful planning and efficient strategies for implementation.

#### Occupational Therapy Perspectives

For OTs, it is critical to give utmost importance to performance than hospital-based rehabilitation, thus making TNR feasible for neuro-OTs (43). OTs are meant to assess and therapeutically manage the actual occupational performance of PWNDs like participation in activities of daily living (ADL), work, and leisure in their home/social environment (44). This strategy could help provide need-based, scientific, and therapeutically rigorous OT services using TNR framework in real-life contexts. Given that many occupational therapy assessments are based on function, it is also possible to incorporate real-life functional assessments for PWNDs. It would also provide immense opportunities for OTs to standardize these assessments for neurorehabilitation in the future in such contexts. Similar to the context of the SLPs, OTs are also scarce globally and especially in LMICs. The most frequently cited domains where OTs are scarce are directly related to conditions that predispose neurological disabilities. Therefore, it is of utmost importance that professional resources and expertise like OTs and SLPs must be protected and developed to address the needs of PWNDs through TNR during the COVID-19 pandemic.

### Challenges and Recommendations for the Implementation of TNR Services in LMICs

Though there are several feasible aspects for implementing TNRs for PWNDs, it is also necessary to understand the barriers that need to be considered with caution. Not everything could be feasible, especially considering how rehabilitation services are organized for PWDs in general in LMICs. Therefore, it is highly pertinent to tease out the barriers for TNR services provided to PWNDs in LMICs.

Table 1 depicts the non-feasible aspects of TNR, especially in LMICs. The first and foremost aspect that may not be feasible while implementing TNR in LMICs is the comprehensive and intrinsically detailed neurological evaluation and treatment. This is especially because some of the evaluation and treatment require safe hands-on as well as moving and handling patients, and one cannot do this in TNR. Similarly, neurologists and physiatrists cannot prescribe certain drugs through TNR (45). There are also certain criteria for patient exclusion unless the TNR service is exclusively developed for the requirements. People with severe cognitive-perceptual, emotional, and behavioral issues, young children, and frail elderly patients with silent aspiration, visual, speech, and hearing impairment can most often be excluded from a comprehensive TNR service that may not be available to all in the LMIC context. PWNDs and their caregiver cooperation, privacy, and non-distractible environment are also some key aspects that could prove challenging and non-feasible while providing TNR services.

**Table 1:** Non-feasible aspects of tele-neurorehabilitation.

**Non-feasible aspects of tele-neurorehabilitation**
**Assessment and treatment**
• Comprehensive as well as specific **n**eurological evaluations (**c**ranial **n**erve **e**xamination (V, VI, VII), **c**erebellar symptoms, **h**igher **c**ortical **s**enses, **r**etinal examinations, **v**isual **n**eglect examinations, **g**rading of **m**uscle tone and strength, **d**ynamic **b**alance, **p**assive **r**ange of **m**otion, TCDs, **f**unctional **s**wallow **a**ssessment) • Young children and frail older adults with speech and hearing impairment • **People** with severe cognitive, emotional, or **behavioral** issues • Specific **specialized** therapy techniques that require the therapist to move and handle patients such as neurodevelopmental therapies • Safe swallowing therapy as well as a safe, comprehensive cognitive-communication therapy • Prescription of **s**chedule X drugs • Adherence to professional, **l**egal, and ethical standards • Prior TNR training and/or experience • Patient cooperation • Non-distractible environment • Private space • The objectivity of the assessment and treatment

From the perspective of the TNR service providers, it is imperative that one needs prior training and sufficient experience in using TNR to deliver neurorehabilitation. This particular aspect is taken for granted since most providers assume that using a smartphone or computer can qualify them to deliver TNR services during the pandemic. It is also important that the neurorehabilitation providers do not lose objectivity of the evaluation and treatment while delivering TNR interventions since there is a high possibility for this to happen in the LMIC contexts. Despite the non-existence of any regulatory framework, the neurorehabilitation provider must maintain their highest legal, professional, and ethical standards to deliver safe and effective rehabilitation.

There are three kinds of clear-cut challenges to the implementation of TNR services for PWNDs in LMICs. They are the scientific, technological, and administrative aspects of implementing TNR. Table 2 provides more details of these aspects. From a scientific perspective, delivering TNR services requires specific competencies, and those competencies are not necessarily taught or achieved by neurorehabilitation professionals exclusively in the pre-pandemic times (46). However, competencies for delivering TNR services play a crucial role in making appropriate professional judgments and decisions for the patients. Lack of specific training and education to achieve such competencies within the curriculum of these professionals is a huge challenge (47). Similarly, striking a balance between patient autonomy and non-maleficence in decision making is very difficult when there is no evidence for such decisions in LMICs. TNR services are just emerging to bridge the gaps in access in LMICs and may not hold the same evidence for treatment delivered in person.

**Table 2:** Barriers to implementation of TNR services in LMICs.

**Barriers to implementation of TNR services**		
**Scientific**	**Technical**	**Administrative**
Competencies	Resources	Feasibility
Ethics	Skills	Acceptability
Quality	Infrastructure	Governance
Evidence	Innovations	TNR literacy

Delivering TNR services has become a necessity in the pandemic situation. However, it must not compromise safe, effective, and good quality patient care that the patient may have received otherwise (36). Obtaining consent for the provision of therapeutic interventions through TNR services is not as easy as doing it in person. PWNDs and their primary caregivers must be thoroughly informed about the pros and cons of the decisions or choices available for treatment, enabling them to consent (48, 49). Given that neurorehabilitation professionals are accountable and responsible for their patients, due considerations must be provided to the scientific, professional, and ethical aspects of delivering TNR services for PWNDs.

There are several technological challenges in delivering TNR services. Aspects such as availability of telecommunication technologies such as computers, tablets, and smartphones; technological access features such as connectivity, bandwidth, data storage and management, server capacity, synchronization, and network; skilled workforce to synergistically support the implementation of TNR services; and the infrastructure for hosting and delivering TNR services for PWNDs must be ensured before embarking into service provision through TNR (50). These aspects require tremendous resources in terms of both funding and technology. It also requires skilled telecommunication experts to work with neurorehabilitation experts to innovate TNR interventions that are safe, effective, and of high public health value.

Additionally, from the perspective of the TNR service providers, it is imperative that one needs prior training and sufficient experience in using TNR to deliver neurorehabilitation. This particular aspect is taken for granted since most providers assume that using a smartphone or computer can qualify them to deliver TNR services during the pandemic. It is also important that the neurorehabilitation providers do not lose objectivity of the evaluation and treatment while delivering TNR interventions since there is a high possibility for this to happen in the LMIC contexts. Despite the non-existence of any regulatory framework, the neurorehabilitation provider must maintain their highest legal, professional, and ethical standards to deliver safe and effective rehabilitation.

Even if the scientific and technological challenges are addressed, administering TNR services is an immense strategic challenge. TNR service itself is an innovative intervention that must be feasible for implementation. In LMICs, much of the health and rehabilitation services is accessed through the private sector; hence, ensuring uniformity in the services and standards similar to the public run services may be challenging (51). Rehabilitation facilities can be small, with only an out-patient facility to a comprehensive in-patient facility. Similarly, the neurorehabilitation team can be a small team with a minimum of neurologists and physiotherapists to a comprehensive team with a physiatrist, OTs, SLPs, orthotist, and neuropsychologist in addition to the neurologist and physiotherapist (8). Therefore, implementation of TNR services within this wide range of service structure is highly challenging. It would certainly require a strong system of governance mechanisms to ensure the quality and safety of TNR services delivered in these facilities. To develop or strengthen the existing governance mechanisms, one must have TNR literacy. Without understanding the principles of neurorehabilitation and the application of telecommunication technology in it, it is not possible to govern TNR services in any context.

The same applies to the primary stakeholder of TNR services. PWNDs and caregivers must have TNR literacy or at least be technology literates to optimize TNR services (7). Without this literacy, patients and caregivers might not find value for their time and resources invested and potentially may not accept TNR services for themselves or their loved ones. It is also a crucial challenge to educate all the stakeholders, primarily the patients and their caregivers, about the benefits of TNR in LMICs.

### Recommendations From the IFNR Research Task Force

Given the lack of organized systems in place for the provision of neurorehabilitation services in general, there needs to be sufficient budgetary allocations and a sector-wide approach to the development of policies and systems for the provision of TNR services for PWNDs. These allocations and actions must be from the Department of the Ministry of Health and all other ministries/departments, such as education, technology, telecommunications, and social justice, in a convergent manner (52). By default, the existing pandemic situation provided an opportunity to optimize the technological innovations in health and scale up these innovations to meet the growing burden of neurological disability in LMICs. Thus, this immense opportunity must be tapped to build capacity for safe and effective TNR services provision for PWNDs in these settings.

There have been several assumptions globally when attempting to understand the implications of TNR services. The primary assumption is that everyone has sufficient information and knowledge about both TNR and the COVID-19 pandemic. This assumption gives anyone the leverage to start TNR services. However, this could be detrimental if the science and standards for practice are not evidence-based. In the context of LMICs and the pandemic, the neurorehabilitation team can give due importance to the performance of the neurologically disabled rather than to sophisticated clinical procedures to ensure TNR becomes a reality in these settings. This can enable bridging the gaps in evidence with relevant science and rigor. This subsequently eludes the scientific community to the need for innovations that can connect people and professionals through technology in this pandemic and beyond. The future of neurorehabilitation could radically change, if we utilize these learning from the pandemic period to make TNR services accessible, affordable, and available (53, 54).

## Conclusion

A potential link must be established between the remote TNR services and in-person neurological rehabilitation service provision. This could add value to the health and social care systems that were previously developed for serving the needs of PWND. The link must have due considerations to the needs of the PWNDs. It must also include the needs of the primary caregivers and family who provide continuous support to PWND before, during, and even in the post-pandemic situation. There is a definite implication that this link must strike a balance between access and availability of neurorehabilitation services. This is especially required in LMICs. There is also a need to make accessibility and availability of TNR services consistent across geography, disciplines, and conditions. This is potentially possible by reducing the variation and inconsistencies in terms of the intensity of rehabilitation measured by the dosage, duration, team expertise involved, and goals set and achieved for PWND in LMICs. An amalgamation of the existing system (pre-pandemic) with the innovative TNR systems to support PWND during the pandemic situation could be a feasible solution. This could serve as a potentially possible strategy, even in the future post-pandemic era.

This situation highlights the importance of two key aspects for immediate attention. The first is building the capacity of the patients, caregivers, and families with rigorous evidence-based training using simple protocols and culturally acceptable versions of optimizing TNR services. The training should also be provided to the providers of TNR services especially in terms of safe and effective use of telecommunication technology for neurological rehabilitation. Second, there is a need for immense government support to legitimately develop resources for TNR services such as guidelines and research evidence and address the growing burden of neurological disabilities in LMICs. The IFNR research task force has realized this opportunity to support the PWNDs in need and also the government. It had initiated the development of systematic action plans toward addressing the burden of neurological disabilities. As a first logical step, IFNR aims to develop a national guideline for the provision of TNR in India, and it recommends the same for all LMICs.

## Data Availability Statement

The original contributions presented in the study are included in the article/supplementary material, further inquiries can be directed to the corresponding author/s.

## Author Contributions

On behalf of the IFNR Taskforce, all the authors contributed equally toward the development of this manuscript. All authors agree to be accountable for the content of the work.

## Conflict of Interest

The authors declare that the research was conducted in the absence of any commercial or financial relationships that could be construed as a potential conflict of interest.

## Publisher's Note

All claims expressed in this article are solely those of the authors and do not necessarily represent those of their affiliated organizations, or those of the publisher, the editors and the reviewers. Any product that may be evaluated in this article, or claim that may be made by its manufacturer, is not guaranteed or endorsed by the publisher.

## The Indian Federation of Neurorehabilitation (IFNR) Research Taskforce

Abhishek Srivastava^1^, Neurorehab Specialist and Director, Centre for Physical Medicine and Rehabilitation, Kokilaben Dhirubhai Ambani Hospital and Medical Research Institute, Mumbai, India; Aishwarya Swaminathan^2^, School of Occupational Therapy, D. Y. Patil University, Navi Mumbai, India; Manigandan Chockalingam^3^, Lecturer in Occupational Therapy, National University of Ireland Galway, Galway, Ireland; Murali K. Srinivasan^4^, Faculty of Occupational Therapy, Meenakshi Academy of Higher Education and Research, Chennai, India; Nirmal Surya^5^, Chairman Surya Neuro Centre Mumbai, President Indian Federation of Neurorehabilitation (IFNR), Mumbai, India; Partha Ray^6^, Consultant Neurologist and Clinical Neurophysiologist National Health Services England, The Walton Centre Liverpool & National Professor of Neurology, Liverpool, United Kingdom; Prasanna S. Hegde^7^, Speech-Language Pathology, Program Director, Speech and Swallow Specialist, Deglutology and Speech-Language Pathology, HCG Hospital, Bangalore, India; Preetie Shetty Akkunje^8^, Senior Research Fellow, Department of Speech Pathology and Audiology, National Institute of Mental Health and Neurosciences, Bangalore, India; Sanjivani Kamble^9^, Dr. D. Y. Patil College of Physiotherapy, Dr. D. Y. Patil Vidyapeeth, Pune, India; Sonal Chitnis^10^, Assistant professor, Bharati Vidyapeeth (Deemed to be) University, School of Audiology Speech-Language Pathology, Pune, India; Sureshkumar Kamalakannan^11^, South Asia Centre for Disability Inclusive Development and Research (SACDIR), Public Health Foundation of India, The Indian Institute of Public Health Hyderabad, Hyderabad, India; Suvarna Ganvir^12^, Department of Neuro physiotherapy, Dr Vitalrao Vikhe Patil Foundation's College of Physiotherapy, Ahmednagar, India; Urvashi Shah^13^, Consultant Neuropsychologist, Department of Neurology, King Edward Memorial and Global Hospitals Mumbai, Mumbai, India.
